# A Retrospective Analysis of Wildlife Rehabilitation Trends in Lithuania over Two Decades

**DOI:** 10.3390/ani16081210

**Published:** 2026-04-16

**Authors:** Aistė Stankūnaitė, Vytautas Ribikauskas, Justina Morkūnaitė, Jūratė Kučinskienė

**Affiliations:** 1 The Faculty of Veterinary Medicine, Lithuanian University of Health Sciences, 47181 Kaunas, Lithuania; vytautas.ribikauskas@lsmu.lt (V.R.); justina.morkunaite@lsmu.lt (J.M.); jurate.kucinskiene@lsmu.lt (J.K.); 2 The Wildlife Rescue Centre, Lithuanian University of Health Sciences, 54317 Naujieji Muniskiai, Lithuania

**Keywords:** wildlife, rehabilitation, wildlife threats, human–wildlife coexistence, Lithuania

## Abstract

The primary aim of wildlife rehabilitation centres is the release of treated animals back into the wild after treatment. Data collected during rehabilitation can provide valuable insights into current trends and can help with conservation strategies aiming to reduce human-related negative impact. This review analyses records from past and currently operating wildlife rescue and rehabilitation centres in Lithuania over a 23-year period. Data were compiled for 7847 individual animals representing 216 species of birds, mammals, and reptiles. The study evaluates patterns of seasonal admission, taxonomic composition, known causes of injury or admission, and rehabilitation outcomes. The results showed that 83% of cases involved birds, mammals comprised 16%, and reptiles were recorded only infrequently (0.52%). Admissions peaked in summer, comprising 42% of all cases. Injuries of unknown origin were the most common (55%). Among cases with identified causes, a substantial proportion were associated with human activities, including road accidents (5% of all cases), collisions with anthropogenic structures (4%), and attacks by domestic cats or dogs (3%). The presence of a specialized rehabilitation centre, together with active public involvement in the rescue of injured wildlife, contributes to release rates reaching approximately 30% of admitted animals. Datasets such as those analyzed in this study may contribute to improved preparedness for managing unavoidable human–wildlife interactions in the future.

## Simple Summary

Wildlife rehabilitation centres aim to treat injured or orphaned wild animals and return them to their natural habitats. In this study, records from past and currently operating wildlife rescue and rehabilitation centres in Lithuania were reviewed over a 23-year period to evaluate trends in wildlife admissions and outcomes. A total of 7847 individual animals representing 216 species of birds, mammals, and reptiles were included in the analysis. Birds dominated the cases (83%), mammals made up 16%, and reptiles were rare, accounting for just 0.52%. Seasonal patterns showed a clear peak during the summer months (42% of all admissions). Injuries of unknown origin were most frequently recorded (55%); however, among cases with identified causes, many were associated with human activities. The most common anthropogenic factors included road traffic accidents (5% of all cases), collisions with human-made structures (4%) and attacks by pets (3%). Overall, approximately 30% of admitted animals were successfully rehabilitated and released back into the wild. The findings highlight the importance of specialized rehabilitation centres and active public involvement in wildlife rescue. Long-term datasets such as these provide valuable information for understanding human–wildlife interactions and can support the development of strategies to reduce negative anthropogenic impacts on wildlife populations.

## 1. Introduction

The relationship between humans and wildlife is complex, emerging from a long history of interaction [1]. Research on human–wildlife contact has traditionally emphasized negative outcomes such as crop depredation, livestock predation, and risks to human safety [2,3]. More recent studies, however, conceptualize coexistence as a reciprocal process. Humans and wildlife share increasingly overlapping spaces and adjust their behaviours in response to one another in ways that mitigate perceived risks [4,5]. This shift reflects a broader movement in this field of study from a conflict-centric point of view towards a more cooperative framework [3].

Anthropogenic changes to landscapes—urban expansion, intensifying agricultural practices, fast spread of transportation infrastructure—have increased the frequency of encounters between people and wild animals [6]. These interactions present both substantial challenges and emerging opportunities. On one hand, rising road traffic, pollution, and habitat fragmentation have contributed to elevated rates of wildlife injury and orphaning, with significant consequences for animal welfare [7,8]. On the other hand, growing public engagement can lead to the development of community-based initiatives and strengthened regulatory protections for wildlife [9].

In human interactions with wild animals, rehabilitation centres play a crucial role in promoting animal welfare and supporting conservation efforts. Across Europe, although the precise number is unknown, numerous centres provide medical care, shelter, and eventual release for injured or displaced wild animals [10,11,12].

Wildlife rehabilitation is defined as ‘the treatment and temporary care of injured, diseased, and displaced indigenous animals, and the subsequent release of healthy animals to appropriate habitats in the wild’ [13]. But work in centres extends beyond rehabilitation: many also monitor populations, contribute to research, and educate the public about conservation [14].

There are also broader debates about whether rehabilitation meaningfully affects species conservation, despite its clear importance for individual animal welfare, compared to habitat protection and preventive measures [15]. Wildlife rehabilitation typically has limited direct effects on population growth for most species, especially those with high reproductive rates. However, for species with low reproductive rates and high adult mortality, rehabilitation can help stabilize or recover declining populations, especially when injury rates are high and rehabilitation is combined with other conservation actions [16,17].

The precise number of wildlife rehabilitation centres remains unclear because each country maintains its own database and policies concerning wildlife. Furthermore, rescue operations may be carried out by various organizations, not all of which call themselves rescue centres [18]. In Europe, some countries have well-developed strategies for the rescue of wild animals. France has over 45 licensed centres for the rehabilitation of wild birds and other animals [19]; Italy has 97 organizations across the country that are managed by public or private entities in close cooperation with local authorities [20].

Examining recent trends and the role of rehabilitation centres helps assess how society can reduce negative impacts and support sustainable coexistence with wildlife [10].

### Lithuanian Context

Over the past three decades, the Baltic States have seen both declines and recoveries in mammal populations. In Lithuania, regional extinctions (e.g., European mink (*Mustela lutreola*), garden dormouse (*Eliomys quercinus*)), and declines in small insectivores (shrews (*Soricidae*), pond bat (*Myotis dasycneme*)) and herbivores (bank vole (*Clethrionomys glareolus*)) have been observed. Boreal specialists like the Siberian flying squirrel (*Pteromys volans*) are contracting their range [21,22,23]. Ungulate populations (roe deer (*Capreolus capreolus*), red deer (*Cervus elaphus*), moose (*Alces alces*), European fallow deer (*Dama dama*)) continue to show an increasing trend, with roe deer now being the most abundant cervid (Table 1). Large carnivores such as wolves (*Canis lupus*) and lynx (*Lynx lynx*) have also recovered, with lynx numbers quadrupling since 2010 [24,25,26]. Invasive species like American mink (*Neogale vison*), raccoon dog (*Nyctereutes procyonoides*), and raccoon (*Procyon lotor*) have expanded, altering native communities and requiring new management strategies [23,27]. These changes are driven by climate variability, land use, disease outbreaks (notably African swine fever in wild boar (*Sus scrofa*)), and rewilding policies, all of which influence the demand for wildlife rehabilitation and the types of cases encountered [28,29].

African swine fever had a major impact on wild boar populations in Lithuania, leading to population declines and increased surveillance. The disease remains endemic, posing ongoing risks to both wild and domestic animals and complicating wildlife management and rehabilitation efforts [28,31,32]. Urbanization and increased road traffic have led to a rise in wildlife–vehicle collisions, especially involving roe deer and moose [33]. COVID-19 lockdowns temporarily altered these patterns, but the overall trend is upward, highlighting the need for management strategies and public awareness to reduce injuries and fatalities among both wildlife and humans [34].

Bird populations in Lithuania are experiencing shifts in abundance and distribution, largely influenced by global climate change [35,36]. Many species are moving their ranges northward or eastward, with rare and endangered birds particularly affected. While some species benefit from these changes, others face population declines or increased vulnerability [37]. For example, the lesser spotted eagle (*Clanga pomarina*) population declined by 26% between 1980 and 2006 [35], but in recent years, thanks to conservation efforts, Lithuania has become the global population stronghold of this raptor. An estimated 1900–2900 breeding pairs are present in the country [38].

Lithuania is a vital link for several threatened European bird species during migration. The White and Baltic Sea migration route passes through the country, with the Curonian Spit serving as a major migratory corridor for over 200 bird species, including many threatened ones [39]. Mires and wetlands in eastern Lithuania are critical for rare and vulnerable species, providing breeding and stopover habitats [40,41,42] (Table 2).

Habitat loss, climate change, and infrastructure (e.g., wind farms) threaten migratory birds along their routes [44,45]. Therefore, alongside species conservation and habitat restoration, it is important to have well-equipped, specialized wildlife rescue centres that can provide urgent assistance and ensure appropriate standards of animal welfare for injured animals of different species.

In the Baltic region, wildlife rehabilitation centres and data about wildlife rehabilitation are notably scarce. Volunteers and individual organizations undertake the majority of wilderness conservation efforts.

The Baltic Sea Animal Rehabilitation Centre (located in Klaipėda, Lithuania) plays a crucial role in rescuing, rehabilitating, and releasing marine animals, especially grey seals, which are impacted by human activities and environmental stressors [46]. The first specialized rescue centre for terrestrial wild animals in Lithuania was opened recently, in 2024.

To the best of the authors’ knowledge, this is the first long-term study to present wildlife rehabilitation data from Lithuania, allowing comparison with similar international studies. The goal is to gain a more complete understanding of admission trends, which can improve our understanding of changes in species and ecosystem health and animal welfare. We examine how the number of reported cases varies over time, both seasonally and throughout the study period, note different species and identify the main reasons for admission.

## 2. Materials and Methods

### 2.1. Location

For this study, wildlife admission data were compiled from three principal wildlife rehabilitation facilities operating in Lithuania during different time periods. All wildlife rescue facilities are located in Kaunas, the country’s second-largest city.

The first facility, operating from 2001 to 2008, was run by a non-governmental organization and primarily treated animals from nearby regions. Wild animals were also delivered by members of the public and collected from other locations across Lithuania. The rehabilitation centre was based within the Veterinary Academy, providing access to specialized equipment for the treatment of diverse wildlife species, with qualified veterinarians available on call.

Following its closure, wildlife rescue activities were continued by a second organization at different premises between 2012 and 2017. Unlike its predecessor, this organization primarily responded to incident reports and collected animals for treatment directly from the field with a smaller team.

In 2024, the Lithuanian University of Health Sciences Wildlife Rescue Centre was established as the first specialized, fully equipped national facility, staffed by trained professionals and capable of responding to calls and retrieving wildlife throughout Lithuania on a 24 h basis.

These circumstances explain both the temporal gaps in the dataset and the substantial increase in the number of animals admitted in recent years, reflecting improved resources and the growing awareness and development of wildlife rescue services.

### 2.2. Data Collection

Most of the records (74%) existed only in paper form and therefore had to be digitized into spreadsheets prior to analysis. In total, 8114 records were collected. A portion of these had to be excluded because they described exotic animals, domestic species that had accidentally entered the wild, or cases lacking essential information. The final dataset comprised 7847 cases.

Over the two decades covered by this study, the admission protocols and the type of information recorded changed. However, the following data were generally (though not always) available and formed the basis of the analyses: date of admission, location and circumstances of discovery, species and subspecies, estimated age, results of the initial clinical examination upon arrival, diagnosis, treatment or husbandry information during rehabilitation, and outcome. Because species-level categorisation would have resulted in small sample sizes for many taxa, broader grouping into three major classes—birds, mammals, and reptiles—was used to facilitate more meaningful comparisons.

### 2.3. Reasons for Admission and Diagnoses

The greatest variability in the clinical records was found in two sections: circumstances of admission and specific diagnoses. To standardize the dataset, these variables were grouped into summary categories based on those most used in the scientific literature.

The cause of injury and the reason for admission were determined based on the available circumstances. As obtaining an accurate history for wild animals is particularly challenging, both the quantity and reliability of information generally depend on the individual who found the animal, especially when the animal is delivered directly to the centre and the veterinarian has no opportunity to examine the site where it was discovered. All available information is recorded by the rehabilitation centre staff in the animal’s admission record.

When an animal is found already injured and the circumstances of the incident are unclear, the precise cause of injury often remains undetermined. Based on either confirmed or presumed causes of debilitation, cases are classified as anthropogenic (e.g., entanglement in fishing line, vehicle collisions, prolonged captivity) or natural (e.g., nestling fall, disease, predation).

If an animal was admitted to the centre in a non-critical condition, a clinical diagnosis was established in all three wildlife rehabilitation centres by a licensed veterinarian following a clinical examination. Where necessary, the clinical assessment was supplemented with additional diagnostic procedures, including radiography, ultrasonography, bacteriological or virological testing, and post mortem examination in cases that remained inconclusive. Diagnoses were grouped into the following categories: clinically healthy, broken bones, exhaustion, head injury, infectious disease, wounds, bruising, other, and unknown. The diagnosis “clinically healthy” was used when the animal presented without any evident abnormalities and exhibited natural, species-specific behaviour. The category “exhaustion” was used as an operational term to describe animals presenting with generalized weakness, lethargy, and poor body condition, without evidence of specific trauma or diagnosed disease. Criteria were based on clinical assessment at admission and varied slightly across taxa.

### 2.4. Statistical Analysis

Cleaned data for were imported from Microsoft Excel into IBM SPSS Statistics for Mac OS, version 30.0 (IBM Corp., Armonk, NY, USA). A chi-square goodness-of-fit test was used to examine whether the distribution of animals in different taxonomic groupings was equal and to evaluate if season affected the distribution of species. The threshold for statistical significance was *p* < 0.05. Descriptive statistics were computed and are presented as the mean ± standard deviation for annual intake and duration of captivity.

## 3. Results

### 3.1. Annual Trends

**Figure 1:**
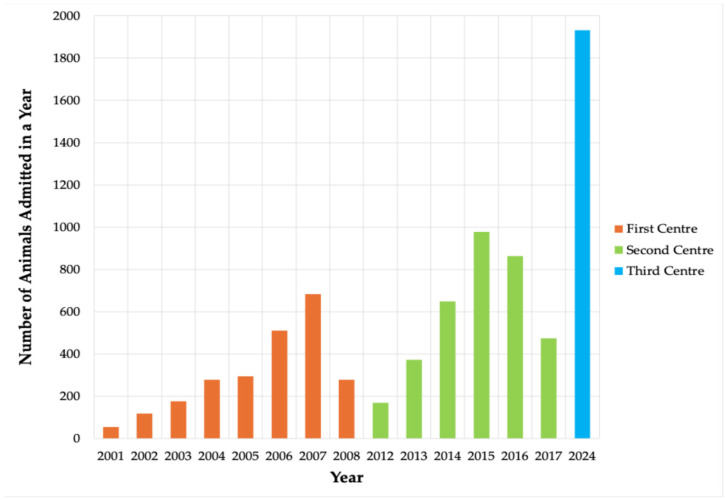
Annual number of animals admitted to the wildlife care centres from 2001 to 2024.

Between 2001 and 2024, a total of 7837 wild animals from across Lithuania were admitted. The annual number of admissions exhibited substantial interannual variability, with gradual increases during the early 2000s, a pronounced peak in 2007, and notable fluctuations throughout the study period. The first facility, established in 2001, recorded the lowest annual intake (n = 55), whereas admissions rose sharply in later years, culminating in the highest recorded value in 2024 (n = 1931), representing a 36-fold increase. As illustrated in Figure 1, this rise was not linear, as periodic declines corresponded to the closure of one facility and the subsequent establishment of another. Across the entire period, the mean annual intake was 522 (SD ± 475.88) cases.

### 3.2. Taxonomic Groups

Animals admitted to rehabilitation were classified into three categories: birds, mammals, and reptiles. Birds represented majority of cases, accounting for 83% (n = 6427). Mammals comprised 16% (n = 1258), while reptiles were recorded only rarely, representing 0.52% (n = 40). The distribution of admissions differed significantly among taxonomic groups (χ^2^(2) = 8931.5, *p* < 0.001).

### 3.3. Seasonal Variation in Admissions

**Figure 2:**
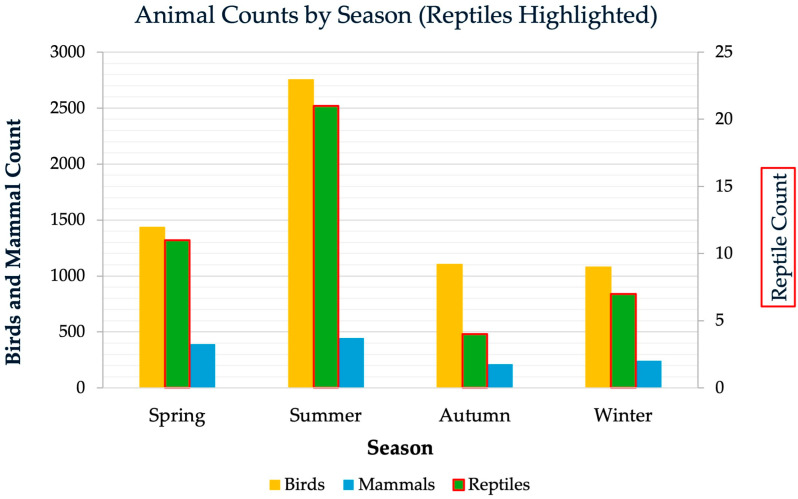
Seasonal distribution of animal admissions, separated by taxonomic group. Due to low numbers, reptile counts are emphasized using a secondary y-axis.

A pronounced seasonality is observed in Lithuania, which may influence the number of cases. Seasonal distribution of wildlife admissions was evaluated using predominant seasonal classification: spring (March–May), summer (June–August), autumn (September–November), and winter (December–February) (Figure 2). Apparent seasonal variation was noted, with most cases recorded in summer, which accounted for 42% of all admissions (n = 3226), followed by spring at 24% (n = 1840). Autumn (17%, n = 1325) and winter (17%, n = 1334) showed substantially lower and nearly equivalent numbers. The association between season and admission counts was statistically significant (χ^2^(6) = 50.55, *p* < 0.001). These results indicate that wildlife admissions are not evenly distributed throughout the year and vary significantly by season across all animal groups. When separated by taxonomic groups, birds, mammals, and reptiles exhibited distinct seasonal patterns: Bird and mammal admissions peaked sharply in summer, with elevated numbers also in spring, while both autumn and winter contributed far fewer cases. Reptile admissions followed a similar pattern, reaching their highest values in summer, but occurred at much lower absolute numbers and comprised less than 1% of all admitted animals.

**Figure 3:**
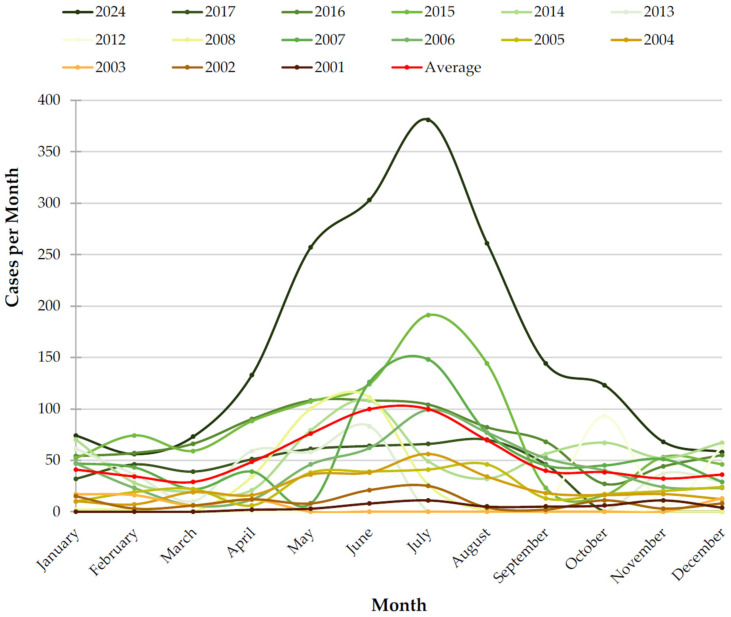
Monthly distribution of wildlife admissions from 2001 to 2024.

The monthly distribution of wildlife admissions from 2001 to 2024 (Figure 3) reveals a strong and consistent pattern. Admissions typically begin to rise in early spring, reaching a pronounced peak in June (n = 1196) and July (n = 1197), each accounting for 16% of all recorded cases. In contrast, the lowest monthly proportions were observed in March (4%, n = 347) and November (5%, n = 387). Annual time-series lines, with darker colours representing more recent years, show substantial interannual variability in both the magnitude and timing of these peaks. Despite this variability, the multi-year average (red line) clearly highlights a predictable mid-summer surge followed by a steady decline toward winter.

### 3.4. Species Admitted

The white stork was the most commonly admitted species (n = 1246) (Table 3), followed by the mute swan (n = 783) and the rock dove (n = 663). Among mammals, the roe deer (n = 415), European hedgehog (n = 270), and red fox (n = 161) were the most frequently presented. Reptiles were absent from the top-listed species.

**Figure 4:**
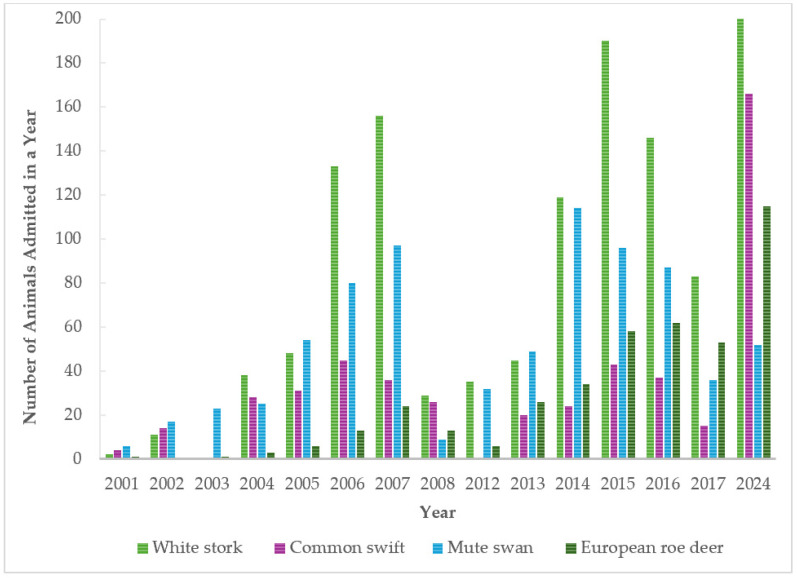
Yearly distribution of four most common species admissions from 2001 to 2024.

Annual counts of the four most common species (white stork, common swift, mute swan, and European roe deer) were recorded between 2001 and 2024 (Figure 4). Overall, the white stork and European roe deer exhibited clear long-term increases, while the common swift showed marked interannual variability with a pronounced rise in 2024. In contrast, mute swan numbers peaked in the mid-2010s and subsequently declined, indicating a different population dynamic.

The dataset (Table 4) presents records of wildlife cases across multiple taxa, with birds comprising the majority of admissions. Waterfowl and other large-bodied bird species were particularly prominent, led by the mute swan (10%) and the white stork (16%). The rock dove (9%) is in a separate category of *Columbiformes*. Among smaller-bodied bird species, the common swift (6%) and hooded crow (5%) were most frequently represented. Mammals accounted for a smaller part, with the roe deer (5%) and European hedgehog (4%) being the most common. Raptors were less frequent, with the common buzzard (3%) as the leading representative.

### 3.5. Age

Animals were classified as either adults or juveniles; age could not be determined or was not recorded for 451 cases (6%). Across all taxonomic groups, adults represented most of the admissions, averaging approximately 72% of all individuals. Mammals showed the highest proportion of adults (77%), while birds and reptiles exhibited similar juvenile proportions of roughly 27–28%. Overall, the dataset is dominated by adult individuals across taxa, with juveniles comprising only about one quarter of recorded cases.

### 3.6. Time Spent in Rehabilitation

The duration of captivity ranged from 0 to 341 days for birds, 0 to 180 days for mammals, and 0 to 36 days for reptiles. On average, animals spent 7.7 days (SD ± 26.33) in rehabilitation care facilities. However, this estimate is not entirely precise, as 30% (n = 2291) of all cases involved animals that remained for 0 days—either due to immediate euthanasia following examination or because clinically healthy individuals had been admitted unnecessarily and were released back into the wild the same day. Excluding animals with a length of stay of 0 days, birds spent an average of 16 days in rehabilitation, whereas mammals and reptiles spent an average of 18 days. The mean was also distorted by some particular species, such as white storks, which, if admitted in autumn after the migration period, would remain in captivity until the following spring.

### 3.7. Reasons for Admission

Causes of admission were identified in 4423 cases—3656 birds, 731 mammals, and 36 reptiles brought to rescue (Table 5). Across all taxa, birds were the majority (83%), followed by mammals (17%) and reptiles (0.8%). The leading cause of admission across taxa was unknown (55%), followed by orphaned (20%). Mammals exhibited a markedly higher incidence of road-traffic-related admissions (18%) compared to birds (2%) and reptiles (11%). Anthropogenic structural causes (entrapment and intrusion into premises) were also more common in mammals (15% combined) than in birds (4%). Several causes were exclusively avian, including migration timing failures (2%), ice entrapment on frozen bodies of water (2%), and electrocution (1%). The rarity of firearm injuries (0.2%) and fire-related admissions (0.1%) demonstrates the low prevalence of direct persecution and environmental disasters in this dataset.

### 3.8. Diagnoses

Diagnoses were recorded for 3947 wildlife admissions, comprising 3291 birds (83%), 616 mammals (16%), and 40 reptiles (1%) (Table 6). Clinically healthy individuals represented the largest category overall (39%; n = 1520), forming the highest proportion within each taxonomic group (39% of birds, 37% of mammals, and 33% of reptiles).

Traumatic injuries constituted a major share of diagnoses. Broken bones were the second most common category (22%), recorded in 23% of birds, 18% of mammals, and 18% of reptiles. Soft-tissue trauma, including bruising, accounted for 10.0% of admissions, with comparable proportions among birds (10%) and mammals (10%). Wounds were documented in 5% of individuals, occurring at similar frequencies in birds (5%) and mammals (5%), with reptiles accounting for 5% of wound cases.

Non-traumatic conditions were less frequently observed. Exhaustion (defined as generalized weakness and poor condition) accounted for 8% of all diagnoses and was proportionally more common in reptiles (18%) compared with birds (8%) and mammals (8%). Head injuries (3%) occurred primarily in birds (3%) and mammals (4%) and were not recorded in reptiles. Infectious diseases comprised 3% of cases, with mammals (7%) showing a higher prevalence than birds (2%). No reptiles were admitted for infectious conditions.

Additional categories included other diagnoses (7%), which were proportionally highest in reptiles (20%), likely reflecting diverse or infrequently encountered conditions within a small sample, and unknown diagnoses (5%), distributed similarly across taxa.

### 3.9. Outcome

**Figure 5:**
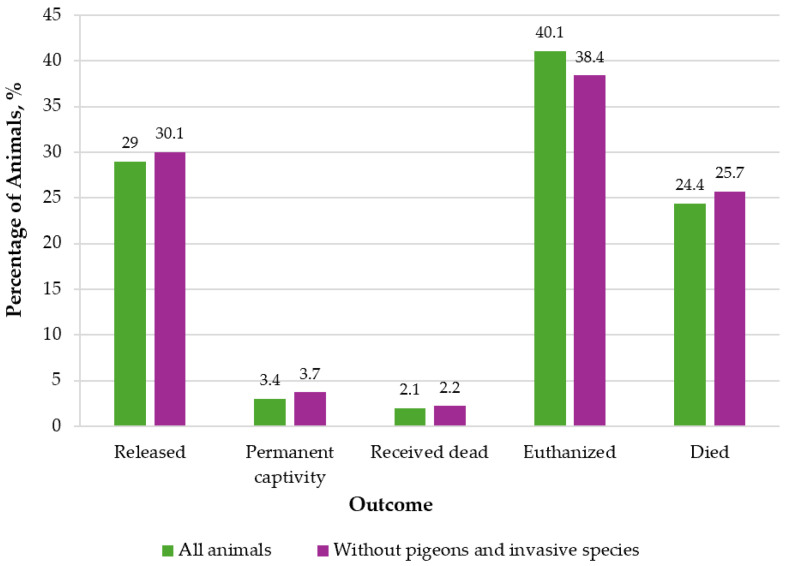
Comparison of wildlife admission outcomes for all animals versus the subset excluding pigeons and invasive species. Bars represent the number of individuals in each outcome category.

Outcome was recorded for 6655 animals (85%). Case outcomes were categorized as release, permanent captivity, euthanasia, or death during examination or treatment (Figure 5). Of the 6655 animals included in the dataset, nearly one-third were successfully returned to the wild (1932; 29%); a small proportion required permanent captivity due to non-lethal yet irreversible impairments (227; 3%). An additional 137 individuals (2%) were received dead on arrival.

Negative outcomes constituted most of the admissions. Euthanasia was the predominant outcome (2734; 41%), followed by animals that died during care (1625; 24%). This pattern highlights the high proportion of cases admitted with injuries or conditions incompatible with recovery.

When pigeons and other invasive species were excluded, the remaining dataset (n = 5959) showed a similar overall structure of outcomes, although shifts in magnitude and proportion were observed. In this subset, 1791 animals were released (30%), 220 required permanent captivity (4%), and 131 were received dead (2%). While euthanasia remained the most frequent outcome (2285; 38%), both the number and proportion of euthanized individuals declined relative to the full dataset. Deaths during care accounted for 1532 cases (26%). These changes indicate that invasive species constitute a disproportionately large share of animals requiring euthanasia.

## 4. Discussion

Wildlife rescue centres play a crucial role in protecting animal welfare, promoting conservation efforts, contributing to scientific research, and raising public awareness. They act as an important link between people and nature, working to reduce the harmful effects of human activities on wildlife and their habitats [20]. Information gathered in rescue centres reveals trends in wildlife health and possible threats (such as vehicle collisions, domestic animal attacks, and disease) [12,47].

Although the number of articles on the success of wild animal rehabilitation has increased substantially over the past decade, such data remain severely lacking in the Baltic region. Recent studies on rehabilitated grey seals in the Baltic Sea show promising post-release survival and behaviour [46], but broader, systematic data on terrestrial wildlife rehabilitation in Lithuania remain limited. This study represents the first national, long-term analysis of wildlife rescue data in Lithuania, derived from admission records from three wildlife rescues.

Our data, and therefore our conclusions, are constrained by which animals are brought to rehabilitation facilities. These admissions do not necessarily represent the broader wildlife population because various biases shape which species and conditions are reported. Human perceptions, such as viewing young or certain species as vulnerable, influence which animals are rescued, while others may be ignored or left in the wild [48]. Records are dominated by certain species, often those that are more visible, accessible, or elicit stronger human concern (e.g., birds, hedgehogs, common mammals) [14,49]. Rare, elusive, or less fascinating species are underrepresented. Animals near homes, roads, and urban areas are also overrepresented, as are conditions easily noticed by people compared to those occurring in remote habitats [48].

### 4.1. Annual Trends

Since 2001, the number of admitted wild animals in Lithuania has increased 36-fold, which may suggest heightened public awareness [50,51]. The substantial increase in admitted animals observed in 2024, following the opening of a specialized centre, highlights its necessity. Appropriate equipment, trained personnel, national-level outreach, and integration into communication networks have helped the centre reach a larger portion of the public and improve responses to wildlife welfare emergencies [52]. These figures are not yet final and continue to rise, a trend that is likely to persist for some time.

### 4.2. Seasonal Variation in Admissions

Admissions exhibited pronounced seasonality, with a peak during the summer months followed by a decline in winter. During summer, juvenile animals are the most frequently noticed by the public, with bird nestlings and fledglings being particularly common. This contributes to increased general notice of their injuries and a higher likelihood of the public seeking assistance from rescues [49,53].

Multiple studies from different regions consistently report that wildlife rescue admissions are also highest in summer, followed by spring. For example, a 10-year study in the UK found that 49% of wildlife admissions occurred in summer, with another 26% in spring [11]. Similar patterns are observed in Australia and Italy, where rescue numbers are lowest in winter and highest in spring and summer [8,20].

This seasonal pattern is also consistent with Lithuania’s position along a migratory flyway and its importance as a breeding ground for both migratory and resident bird species. The Curonian Spit and adjacent Baltic coastline are among the most significant bird migration corridors in Europe. Over 3.2 million birds of 202 species were ringed on the Curonian Spit between 1956 and 2020, providing extensive data on migration routes and population dynamics [39].

### 4.3. Species Admitted

Birds are the most frequently admitted group in many regions, often comprising 60–86% of admissions [49,53]. Our findings fall at the upper end of the reported range, reaching 83%. Mammals typically account for 12–42% of admissions (16% from our data); reptiles and amphibians are less commonly admitted, usually representing less than 5% of cases, though this varies by region. Lithuania, compared with some other countries, has relatively low reptile diversity, which is reflected in a minimal admission rate of 0.52% [11,14,49].

Rescue centres contribute to the conservation of threatened and endangered species by treating and releasing individuals, sometimes including those protected by CITES or on the IUCN Red List [12,20]. European IUCN assessments show that the majority of admitted species are of least concern status, although trends vary: some of the most treated species (e.g., white stork, hooded crow, roe deer) are on the increase, whereas others, such as the common swift (near threatened) and rook (vulnerable), are experiencing declines. Rare and endangered species do appear in wildlife rehabilitation centres, although they do not rank among the most frequently admitted. Roe deer comprise 5% of all cases, making them the most admitted mammal.

These findings highlight the importance of long-term monitoring, particularly for species experiencing regional declines despite relatively common occurrence in the dataset. The prevalence of synanthropic species (e.g., pigeon, house sparrow, red fox) also reflects their frequent interactions with human environments, further underscoring the role of anthropogenic factors in shaping wildlife case distributions and affecting animal welfare [54,55].

### 4.4. Time Spent in Rehabilitation

Most animals spend between 2 and 10 weeks in wildlife rehabilitation centres, with duration influenced by species, cause of admission, and health status [10]. Our estimated mean duration of animals’ stay in care was shorter, averaging up to 8 days. Other authors report that reptiles spend the longest time in care [56]. Our data indicate that reptiles are typically released back into the wild at a similar rate to mammals, but the small sample size of reptiles relative to mammals limits the proportionality of the comparison.

### 4.5. Reasons for Admission and Age

Across all animal groups, the cause of injury most frequently remained unknown (ranging from 43% in mammals to 57% in birds). However, in cases where the cause was identified, human-related threats predominated. These included vehicle collisions, injuries inflicted by cats or dogs, entanglement in discarded fishing lines, and collisions with windows or other human-built structures, together accounting for 21% of cases with known causes. This pattern is consistent with findings reported by other wildlife rehabilitation centres; for example, in Bulgaria, 40% of patients were received with injuries from unknown causes, 18% from anthropogenic causes, 32% from natural factors, and 10% from reintroduction programs [57].

Wildlife and vehicle collisions are a major cause of injury and death for many species, not only in Lithuania, but worldwide. High-traffic and even low-traffic roads can fragment habitats, increase mortality, and isolate populations, with car strikes being one of the most common causes of wildlife admissions to rehabilitation centres. Also, expanding urbanization and road networks lead to habitat loss, fragmentation, and genetic isolation, further threatening wildlife populations [7,8,58].

Parentless juveniles represented the second most common reason for admission, a pattern particularly pronounced among birds. As noted earlier, people often bring young animals to rehabilitation centres believing they need rescuing. In many cases, these juveniles arrive healthy and uninjured, indicating that their parents were likely nearby, and that intervention was unnecessary. However, limited public awareness of wildlife behaviour, combined with human empathy and a strong desire to help, often leads to well-intentioned but unwarranted removals from the wild, which may inadvertently affect the animals’ natural development and welfare. Admissions of orphaned young and injured wildlife are rising, with orphaned animals representing a significant portion of wildlife hospital cases in many different countries [8,10,11].

Many retrospective studies emphasize that standardized and consistent record-keeping is essential for using wildlife rehabilitation centre data to quantify threats and guide mitigation [8,11,59]. However, practices remain inconsistent. For example, a survey in Finland found that only about 20% of rehabilitators kept admission records, raising concerns about data quality and ethical decision-making [60]. In addition, inconsistent coding of causes (e.g., “found”, “unknown trauma”) often leaves many cases without a clear etiology. Implementing standardized history-taking and coded record-keeping in wildlife hospitals and rehabilitation centres would increase the proportion of cases with identifiable causes, improving triage, welfare, and conservation planning.

### 4.6. Diagnoses

Wildlife admissions are often trauma-related conditions with notable differences among taxonomic groups. Across all three animal classes, the most common diagnosis was “clinically healthy,” indicating that many animals were removed from the wild unnecessarily, mostly juveniles. The next two most frequent diagnoses were bone fractures and severe exhaustion. Animals suffering from these conditions are less able to hide or evade threats, making them more visible to people and more likely to be brought to rehabilitation centres, which may explain their high representation in the dataset [14,49].

### 4.7. Outcome

Collectively, the outcome data (Figure 4) demonstrate that although a substantial fraction of admitted animals can be rehabilitated and released, mortality outcomes, particularly euthanasia, represent a major component of wildlife care caseloads. The removal of invasive species from the dataset further underscores their influence on overall outcome distributions.

More than two-thirds of admissions resulted in outcomes other than successful release, highlighting both the severity of injuries and conditions observed in admitted wildlife, as well as the limitations of rehabilitation in cases of extensive trauma or disease. Our outcome data are consistent with reports from other rehabilitation centres [10,11,20,49]. The literature indicates that, unfortunately, admission to a centre often results in euthanasia for a substantial proportion of animals. Euthanasia rates in wildlife rescue typically range from 15% to over 40%, depending on species, injury type, and local protocols. Most euthanasia is performed to alleviate suffering in animals with severe injuries or poor prognosis, highlighting the ethical and practical challenges animal welfare challenges faced by wildlife rehabilitators. Successful release rates also vary widely, with typically only 30–70% of admitted animals, across all ages and species, being returned to the wild.

A substantial proportion of euthanasia cases in the current study can be attributed to invasive species, which are frequently admitted to rehabilitation centres by the public, but, in accordance with Lithuanian legislation, must be euthanized.

Furthermore, a large share of admissions consists of birds with wing or leg fractures, injuries that are particularly challenging to treat successfully. Avian bones are thin, brittle, and often hollow, making them prone to comminuted fractures rather than clean breaks. This complicates stabilization and healing, as fragments are harder to align and fix [61,62]. By the time these birds arrive at the centre, even relatively minor fractures are often complicated, with infections commonly present [63], and compound fractures almost invariably necessitate euthanasia to prevent prolonged suffering and protect animal welfare. Full recovery must restore perfect alignment and function. Even minor malunions or small changes in bone rotation can severely impair flight, making the bird non-releasable [61,64].

This study would benefit from post-release monitoring data, which is essential for evaluating the true outcomes of wildlife rehabilitation. Release alone does not guarantee success, as rates of post-release survival and integration into wild populations often remain unknown [65,66]. A global meta-analysis of 112 studies on birds and mammals found substantial variation in survival during rehabilitation and after release, depending on species, cause of admission, and release conditions, highlighting the need for systematic post-release data to develop effective protocols [67]. However, surveys indicate that only about one-third to half of rehabilitation centres conduct any monitoring, typically for less than six months [65,68]. Available evidence suggests that rehabilitated birds may have lower annual survival than wild conspecifics [69], whereas in some species (e.g., bears, cheetahs, seals, sea turtles, polecats) survival, movements, and even reproduction can be comparable to wild populations. Monitoring methods include banding or ringing, visual resighting, photo-identification, and tracking technologies such as VHF, GPS, or satellite tags. Ethical frameworks recommend minimizing device impacts and clearly defining monitoring objectives [69,70,71,72,73]. Overall, post-release monitoring is critical for assessing survival and behaviour, improving rehabilitation protocols, and ensuring that rehabilitation benefits both animal welfare and conservation.

Evidence-based protocols tailored to each species and situation are essential for improving wildlife survival during rehabilitation and after release [67].

## 5. Conclusions

A diverse range of animal species is admitted to wildlife rehabilitation care facilities, with a predominance of those that are well-adapted to human-dominated landscapes. Although the precise causes of admission are frequently undocumented, cases with identifiable origins indicate that injuries are often the result of direct or indirect human activities. The high rates of euthanasia and mortality among admitted animals suggest that, even after reaching veterinary care, injuries are frequently too severe or too advanced to allow successful treatment within the constraints of species-specific biology and welfare considerations. Preventing such injuries, particularly those attributable to human actions, should therefore be a primary objective.

Systematic collection and analysis of data from wildlife rehabilitation centres can play a crucial role in identifying animal injury patterns and informing prevention strategies. These data are essential for the development of national wildlife management policies and for evidence-based planning of urban and national infrastructure, including transportation networks. Incorporating and adapting successful mitigation measures implemented in other countries is strongly recommended. Such an integrated approach is necessary to promote long-term coexistence between the human population and wildlife.

## Figures and Tables

**Table 1:** Game animal census in Lithuania during the 2014–2015 and 2024–2025 hunting seasons [30].

Species	Year 2014–2015 *n*	Year 2024–2025 *n*
Roe deer (*Capreolus capreolus*)	111,427	162,592
Red deer (*Cervus elaphus*)	30,056	96,937
Eurasian beaver (*Castor fiber*)	44,416	46,755
Wild boar (*Sus scrofa*)	22,325	28,720
Moose (*Alces alces*)	10,903	21,049
European badger (*Meles meles*)	2980	18,874
European fallow deer (*Dama dama*)	2325	13,316

**Table 2:** Key threatened migratory bird species and their habitats in Lithuania. Global status assessment by IUCN Red List (2025) [43].

Species	Status	Key Habitats in Lithuania
Aquatic Warbler (*Acrocephalus paludicola*)	Globally threatened	Wetlands, fen mires
Eurasian Curlew (*Numenius arquata*)	Near threatened	Baltic coast, wetlands
Black Grouse (*Lyrurus tetrix*)	Vulnerable	Eastern mires
Black Stork (*Ciconia nigra*)	Vulnerable	Wetlands, forests

**Table 3:** Species (common and Latin names) with more than 100 identified individuals admitted over the study period. All species included are classified as least concern (LC) in Lithuania.

Common Name	Latin Name	*n*
White Stork	*Ciconia ciconia*	1246
Mute Swan	*Cygnus olor*	783
Rock Dove	*Columba livia*	663
Common Swift	*Apus apus*	489
Roe Deer	*Capreolus capreolus*	415
Hooded Crow	*Corvus cornix*	370
European Hedgehog	*Erinaceus europaeus*	270
Mallard	*Anas platyrhynchos*	230
Common Buzzard	*Buteo buteo*	228
Red Fox	*Vulpes vulpes*	161
Rook	*Corvus frugilegus*	142
Common Gull	*Larus canus*	137
Western House Martin	*Delichon urbica*	133
House Sparrow	*Passer domesticus*	118
Song Thrush	*Turdus philomelos*	109
Tawny Owl	*Strix aluco*	102

**Table 4:** The most frequently admitted wildlife species and their conservation status. The table lists species grouped by major taxonomic categories, along with the total number of admission cases (n), their proportional contribution to all admissions (%), and their current IUCN Red List assessment for Europe (LC = least concern, NT = near threatened, VU = vulnerable, NA = not applicable; arrows indicate population trends: ↑ increasing, ↓ decreasing, – stable or unknown).

Species	Total Number of Cases		IUCN EU Assessment
	*n*	%	
**Songbirds (53 spp.)**	1295	16.8	–
Common swift (*Apus apus*)	489	6.3	NT ↓
Hooded crow (*Corvus cornix*)	370	4.7	LC ↑
Rook (*Corvus frugilegus*)	142	1.8	VU ↓
Northern house martin (*Delichon urbicum*)	133	1.7	LC –
House sparrow (*Passer domesticus*)	118	1.5	LC ↓
Song thrush (*Turdus philomelos*)	109	1.4	LC –
Common starling (*Sturnus vulgaris*)	92	1.2	LC –
Eurasian jackdaw (*Coloeus monedula*)	80	1	LC ↑
Eurasian magpie (*Pica pica*)	63	0.8	LC –
Eurasian jay (*Garrulus glandarius*)	57	0.7	LC –
Eurasian blackbird (*Turdus merula*)	56	0.7	LC ↑
**Wading birds (14 spp.)**	1345	17.4	–
White stork (*Ciconia ciconia*)	1246	16	LC ↑
**Waterfowl and seabirds (31 spp.)**	1341	17.4	–
Mute swan (*Cygnus olor*)	783	10.1	LC ↑
Mallard (*Anas platyrhynchos*)	230	2.9	LC ↓
Mew gull (*Larus canus*)	137	1.8	LC
European herring gull (*Larus argentatus*)	88	1.1	LC ↓
Goosander (*Mergus merganser*)	59	0.8	LC ↑
**Pigeons and doves (4 spp.)**	702	9.1	–
Rock dove (*Columba livia*)	663	8.6	LC ↓
**Mammals (27 spp.)**	1258	16.3	–
European roe deer (*Capreolus capreolus*)	415	5.4	LC ↑
European hedgehog (*Erinaceus roumanicus*)	270	3.5	LC
Red fox (*Vulpes vulpes*)	161	2	LC ↑
Raccoon dog (*Nyctereutes procyonoides*)	90	1.2	NA –
European hare (*Lepus europaeus*)	61	0.8	LC –
Eurasian red squirrel (*Sciurus vulgaris*)	51	0.7	LC
Stone marten (*Martes foina*)	48	0.6	LC –
**Raptors (22 spp.)**	423	5.5	–
Common buzzard (*Buteo buteo*)	228	3	LC ↑
Tawny owl (*Strix aluco*)	102	1.3	LC –
Eurasian sparrowhawk (*Accipiter nisus*)	72	0.9	LC –
Little owl (*Athene noctua*)	49	0.6	LC
**Reptiles/amphibians (8 spp.)**	40	0.5	–
**Woodpeckers (6 spp.)**	102	1.3	–

**Table 5:** Causes of admission for birds, mammals, and reptiles treated at the wildlife rehabilitation care facilities. Values represent the number of individuals per taxonomic group, with percentages indicating the proportion within each group. Labels indicate both absolute numbers and the percentage of total admissions per taxon.

Cause of Admission	Birds	Mammals	Reptiles	Total
Unknown	2072 (56.7%)	312 (42.7%)	17 (47.2%)	2401 (54.5%)
Orphan	802 (21.9%)	91 (12.4%)	0	893 (20.3%)
Road accident	88 (2.4%)	132 (18%)	4 (11.1%)	224 (5.1%)
Got into premises	102 (2.8%)	54 (7.4%)	5 (13.9%)	161 (3.6%)
Collision	138 (3.8%)	17 (2.3%)	0	155 (3.5%)
Attacked by pet (cat or dog)	93 (2.5%)	25 (3.4%)	3 (8.3%)	121 (2.8%)
Trapped in human-made structure	39 (1.1%)	53 (7.3%)	3 (8.3%)	95 (2.2%)
Did not migrate in time	74 (2%)	0	0	74 (1.7%)
Swallowed or embedded fishing line	71 (1.9%)	3 (0.4%)	0	54 (1.2%)
Held in captivity	47 (1.3%)	19 (2.6%)	2 (5.6%)	68 (1.5%)
Ice entrapment on frozen body of water	58 (1.6%)	0	0	58 (1.3%)
Overhead lines	49 (1.3%)	0	0	49 (1.1%)
Attacked by natural predator	15 (0.4%)	18 (2.5%)	2 (5.6%)	35 (0.8%)
Shot	6 (0.2%)	4 (0.5%)	0	10 (0.2%)
Fire	2 (0.1%)	3 (0.4%)	0	5 (0.1%)
Total	3656	731	36	4423

**Table 6:** Distribution of clinical diagnoses among admitted wildlife by taxonomic group.

Diagnosis	Birds	Mammals	Reptiles	Total
Clinically healthy	1282 (39%)	225 (36.5%)	13 (32.5%)	1520 (38.5%)
Broken bones	752 (22.9%)	108 (17.5%)	7 (17.5%)	867 (22%)
Bruising	335 (10.2%)	60 (9.7%)	0	395 (10%)
Exhaustion	253 (7.7%)	46 (7.5%)	7 (17.5%)	306 (7.8%)
Other	209 (6.4%)	51 (8.3%)	08 (20%)	268 (6.8%)
Wounds	152 (4.6%)	33 (5.4%)	2 (5%)	187 (4.7%)
Unknown	154 (4.7%)	27 (4.4%)	3 (7.5%)	184 (4.6%)
Head injury	91 (2.8%)	25 (4.1%)	0	116 (2.9%)
Infectious disease	63 (1.9%)	41 (6.7%)	0	104 (2.6%)
Total	3291	616	40	3947

## Data Availability

The data presented in this study are available on request from the corresponding author.
